# Dystrophin deficiency in canine X-linked muscular dystrophy in Japan (CXMD_J_) alters myosin heavy chain expression profiles in the diaphragm more markedly than in the tibialis cranialis muscle

**DOI:** 10.1186/1471-2474-9-1

**Published:** 2008-01-09

**Authors:** Katsutoshi Yuasa, Akinori Nakamura, Takao Hijikata, Shinichi Takeda

**Affiliations:** 1 Department of Anatomy and Cell Biology, Research Institute of Pharmaceutical Sciences, Faculty of Pharmacy, Musashino University, Nishi-tokyo, Tokyo 202-8585, Japan; 2 Department of Molecular Therapy, National Institute of Neuroscience, National Center of Neurology and Psychiatry, Kodaira, Tokyo 187-8502, Japan

## Abstract

**Background:**

Skeletal muscles are composed of heterogeneous collections of muscle fiber types, the arrangement of which contributes to a variety of functional capabilities in many muscle types. Furthermore, skeletal muscles can adapt individual myofibers under various circumstances, such as disease and exercise, by changing fiber types. This study was performed to examine the influence of dystrophin deficiency on fiber type composition of skeletal muscles in canine X-linked muscular dystrophy in Japan (CXMD_J_), a large animal model for Duchenne muscular dystrophy.

**Methods:**

We used tibialis cranialis (TC) muscles and diaphragms of normal dogs and those with CXMD_J _at various ages from 1 month to 3 years old. For classification of fiber types, muscle sections were immunostained with antibodies against fast, slow, or developmental myosin heavy chain (MHC), and the number and size of these fibers were analyzed. In addition, MHC isoforms were detected by gel electrophoresis.

**Results:**

In comparison with TC muscles of CXMD_J_, the number of fibers expressing slow MHC increased markedly and the number of fibers expressing fast MHC decreased with growth in the affected diaphragm. In populations of muscle fibers expressing fast and/or slow MHC(s) but not developmental MHC of CXMD_J _muscles, slow MHC fibers were predominant in number and showed selective enlargement. Especially, in CXMD_J _diaphragms, the proportions of slow MHC fibers were significantly larger in populations of myofibers with non-expression of developmental MHC. Analyses of MHC isoforms also indicated a marked increase of type I and decrease of type IIA isoforms in the affected diaphragm at ages over 6 months. In addition, expression of developmental (embryonic and/or neonatal) MHC decreased in the CXMD_J _diaphragm in adults, in contrast to continuous high-level expression in affected TC muscle.

**Conclusion:**

The CXMD_J _diaphragm showed marked changes in fiber type composition unlike TC muscles, suggesting that the affected diaphragm may be effectively adapted toward dystrophic stress by switching to predominantly slow fibers. Furthermore, the MHC expression profile in the CXMD_J _diaphragm was markedly different from that in *mdx *mice, indicating that the dystrophic dog is a more appropriate model than a murine one, to investigate the mechanisms of respiratory failure in DMD.

## Background

Duchenne muscular dystrophy (DMD) is an X-linked, lethal disorder of skeletal muscle caused by mutations in the dystrophin gene, which encodes a large sub-sarcolemmal cytoskeletal protein, dystrophin. DMD is characterized by a high incidence (1 in 3,500 boys) and a high frequency of *de novo *mutation [1]. The absence of dystrophin is accompanied by the loss of dystrophin-associated glycoprotein complex from the sarcolemma, leading to reduce membrane stability of myofibers. This dysfunction results in progressive muscle weakness, cardiomyopathy, and subsequent early death by respiratory or heart failure in DMD patients.

For basic and therapeutic studies of DMD, it is very important to perform analysis and evaluation using dystrophin-deficient animal models, such as the *mdx *mouse and dystrophic dog. The *mdx *mouse has been well utilized in many DMD studies, but the murine model shows moderate dystrophic changes unlike severe human DMD [2]. In contrast, golden retriever muscular dystrophy (GRMD) shows similar dystrophic phenotypes to those of human patients: elevated serum CK level, gross muscle atrophy with joint contracture, cardiomyopathy, prominent muscle necrosis, degeneration with mineralization and concurrent regeneration, and endomysial and perimysial fibrosis [3]. Therefore, the dystrophic dog is more suitable than the *mdx *mouse for studies to gain insight into the pathogenic and molecular biological mechanisms of human DMD, as well as for pre-clinical trials [4]. Therefore, we have recently established a colony of beagle-based canine X-linked muscular dystrophy in Japan (CXMD_J_) [5], and have demonstrated that CXMD_J _also exhibited severe symptoms similar to GRMD. To date, we have utilized the littermates of the CXMD_J _colony for pathological [6,7], molecular biological [8], and therapeutic examinations [9] of DMD.

Skeletal muscles are composed of heterogeneous populations of muscle fiber types, which contribute to a variety of functional capabilities. In addition, muscle fibers can adapt to diverse situations, such as aging, exercise, and muscular diseases, by changing fiber size or fiber type composition. Therefore, it is important to analyze fiber types to evaluate the condition of skeletal muscle with disease. Fiber types can be distinguished by biochemical, metabolic, morphological, and physiological properties. One of the most informative methods for identification of fiber types is detection of myosin heavy chain (MHC) [10,11]. Myofibers express various MHC isoforms containing slow (type I), fast (types IIA, IIX, IIB), embryonic, and neonatal forms. MHC expression, however, seems to differ between animal species and muscle types. Three MHC isoforms (types I, IIA, and IIX) have been identified in limb skeletal muscles of human and dog, while the fourth isoform, MHC IIB, is abundantly present in small mammals including mouse [10,11]. In addition, expression profiles of MHCs in dystrophin-deficient muscles have been widely examined in limb skeletal muscles of DMD patients [12] and animal models, such as the *mdx *mouse [13] and GRMD [14], but it has not been fully analyzed in skeletal muscles of a canine model. Furthermore, expanded studies of the diaphragm were restricted to that of the *mdx *mouse [13,15]. Therefore, it is important to perform detailed evaluation of fiber types and fiber sizes in limb skeletal muscles and the diaphragm of CXMD_J _to understand adaptations toward disease by changes in fiber type composition in the skeletal muscles of human DMD.

In this study, to investigate fiber types of myofibers in dystrophin-deficient skeletal muscles of dystrophic dogs, we evaluated the expression profiles of MHCs in tibialis cranialis (TC) muscles and diaphragms of CXMD_J _at various ages, by immunohistochemical and electrophoretic techniques. Briefly, we detected myofibers expressing fast type, slow type, and/or developmental MHCs. In addition, the numbers of fast or slow MHC fibers and the size distribution of these myofibers were analyzed among populations of muscle fibers with or without developmental MHC. The composition of MHC isoforms was also examined in pairs of normal and affected dogs at various ages. This is the first report of evaluation of the detailed distribution of fiber types in TC muscles and diaphragms of dystrophic dogs.

## Methods

### Animals

Experimental dogs were wild-type and dystrophic littermates at ages from 1 month to 3 years, from the beagle-based CXMD_J _breeding colony at National Center of Neurology and Psychiatry (Tokyo, Japan) [5,6]. Within a few days after birth, the genotypes (wild-type, carrier, or dystrophy) of the littermates were determined by a snapback method of single-strand conformation polymorphism (SSCP) analysis [16], and the phenotypes were also confiermed by measuring serum CK level [5]. All animals were cared for and treated in accordance with the guidelines approved by Ethics Committee for Treatment of Laboratory Animals at NCNP, where three fundamental principles (replacement, reduction, and refinement) were also considered. Adult control and CXMD_J _dogs (10 months to 3 years) were analyzed in early experiments (three to six animals). Series consisting of a pair of a normal dog and an affected littermate at ages of 1, 2, 4, 6 months, or 1 year old were examined in subsequent experiments. TC muscles and diaphragms were removed from the dogs after necropsy, in which euthanasia was performed by exsanguination under anesthesia with isoflurane taken to prevent unnecessary pain. TC muscle was used as a representative limb skeletal muscle, and it corresponds to the tibialis anterior muscle in mice and humans. The muscle blocks were divided into pieces and frozen immediately in isopentane pre-cooled with liquid nitrogen.

### Histological and immunohistochemical analysis

Serial transverse cryosections (10 μm thick) were stained with hematoxylin and eosin (H&E), and immunostained using anti-MHC antibodies. Immunohistochemistry was performed as described previously [17]. Cryosections were incubated with the following primary antibodies: mouse monoclonal antibodies against fast type MHC (NCL-MHCf; Novocastra), slow type MHC (NCL-MHCs), and developmental MHC (NCL-MHCd). The primary antibodies were detected using a Vectastain^® ^ABC kit (Vector Laboratories) and then visualized with diaminobenzidine. Images were recorded using a microscope (Eclipse E600; Nikon) equipped with a CCD camera (HV-D28S; Hitachi), and fiber types of individual myofibers from 400 to 1200 per muscle were identified, based on serial sections immunostained with three types of MHC antibodies. Subsequently, the fiber number of each group was counted, and fiber sizes were also measured using Image-Pro Plus (Media Cybernetics). Furthermore, the differences in MHC expression between two groups (normal, dMHC (-) *vs *affected, dMHC (-); affected, dMHC (-) *vs *affected, dMHC (+)), between muscles (TC muscle *vs *diaphragm), or among ages (1, 2, 4, 6 months, and 1 year) were evaluated by Yates's chi-square test.

### Myosin extraction and gel separation

Myosin was extracted on ice for 60 min from cryosections, as described previously [18,19]. MHC isoforms were separated on 8% SDS-polyacrylamide gels containing 30% glycerol, according to the methods described previously [19,20] with some modifications. Briefly, aliquots of 0.4 μg of total protein were loaded in each well of mini-gels (Bio-Rad). Electrophoresis was carried out at 60 V at 5°C for 48 h using upper buffer containing additional 10 mM 2-mercaptoethanol. The gels were stained with silver, and the image was scanned and analyzed using NIH image.

## Results

### MHC expression in TC muscle and diaphragm of adult CXMD_J_

**Figure 1:**
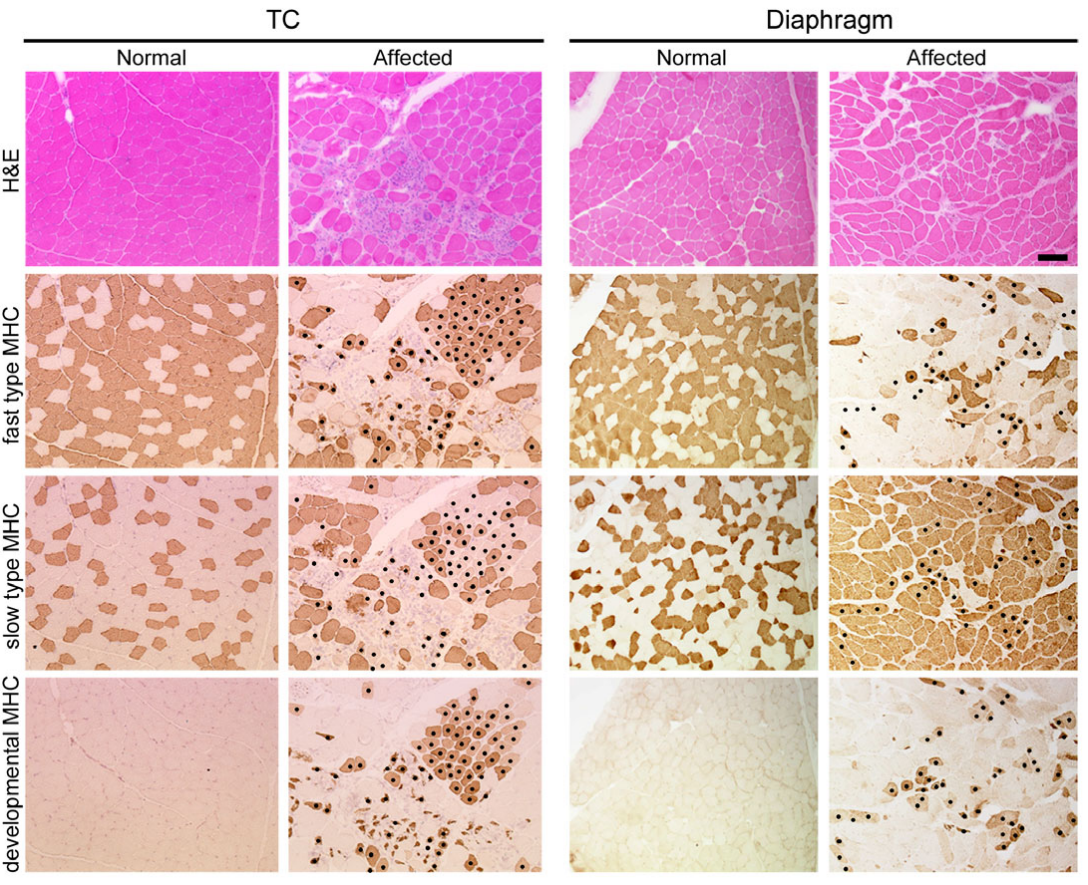
**Representative images of histology (H&E) and expression of fast type, slow type, or developmental myosin heavy chain (MHC) in tibialis cranialis (TC) muscle and diaphragm of a normal (10 months old) or a CXMD_J _dog (11 months old)**. Identical parts of serial cross-sections are shown in longitudinal panels. In panels of affected muscles, dots show the fibers expressing developmental MHC. Bar: 200 μm.

To investigate the relationship between the pathology and fiber types in dystrophic skeletal muscles of CXMD_J_, we first examined histological features and MHC expression in TC muscles and diaphragms of normal and affected dogs at adult stages (10 months to 3 years old) (Fig. 1). In H&E-stained sections, affected muscles exhibited some dystrophic characteristics, such as necrosis, regeneration, cellular infiltration, fibrosis, fiber splitting, and fiber size variation. Especially, clusters of infiltrating cells were prominently observed in TC muscles, while endomysial fibrosis was predominant in diaphragms.

We next detected expression of fast and slow type MHCs for fiber type identification, and further examined developmental MHC, which means neonatal and/or embryonic MHC, as a marker of regenerating fibers (Fig. 1). In TC muscles and diaphragms of adult normal dogs, individual myofibers showed expression of either fast or slow type MHC. In affected TC muscles, the proportions of fast or slow MHC fibers were similar between normal and affected muscles. In addition, large numbers of developmental MHC-expressing fibers were observed in clusters, and many of these fibers co-expressed fast type MHC. In the affected diaphragms, the numbers of fast MHC fibers were much lower than in the normal counterparts, and slow type MHC was expressed in almost all fibers. Furthermore, the numbers of developmental MHC fibers were less than in affected TC muscle, and almost all of these fibers co-expressed slow type MHC, unlike TC muscle. These results indicated that the influences of dystrophin deficiency on MHC expression are significantly different between TC muscle and the diaphragm of CXMD_J_, suggesting that the diaphragm would be more greatly influenced with regard to the composition of fiber types and muscle regeneration than TC muscle.

**Figure 2:**
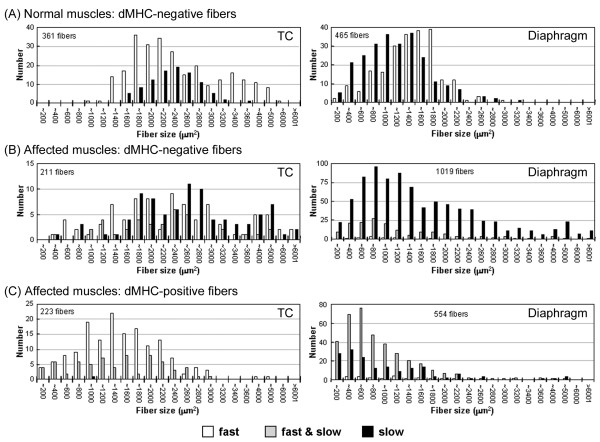
**The size distribution of myofibers expressing fast and/or slow type MHCs in skeletal muscles of a normal (10 months old) or a CXMD_J _dog (11 months old)**. On the basis of expression of fast and slow type MHCs, all fibers within an area of TC muscle or diaphragm of a normal (A) or an affected dog (B, C) were classified into three types of MHC-positive fiber. Furthermore, fast (white), hybrid (gray), or slow MHC myofibers (black) were analyzed among populations of muscle fibers with non-expression of developmental MHC (A, B) or with expression of developmental MHC (C) in terms of fiber numbers (see Table 1) and fiber sizes (A-C). Note that larger sizes of slow MHC fibers were noticeable in populations of muscle fibers expressing fast and/or slow MHC(s) but not developmental MHC of affected muscles (B).

### MHC expression and fiber size distribution

To further evaluate the size distribution of individual myofibers related to MHC expression, we measured transverse areas of all muscle fibers within one area in TC muscle or diaphragm of adult CXMD_J _(Fig. 2 and Table 1). We then analyzed three types of MHC-positive fibers (fast, slow, and hybrid) among populations of myofibers expressing fast and/or slow type MHC(s) together with or without developmental MHC, which were defined as regenerating or non-regenerating fibers, respectively. In non-regenerating fibers of affected TC muscle and diaphragm, the proportion of slow MHC fibers increased and these fibers showed a larger size distribution than those in the normal counterparts, indicating increased number and enlarged fiber size of slow fibers (Fig. 2B and Table 1). Interestingly, fast MHC fibers disappeared in the adult CXMD_J _diaphragm.

**Table 1:** The numbers of myofibers co-expressing fast type, slow type, and/or developmental MHCs in skeletal muscles of a normal (10 months old) or a CXMD_J _dog (11 months old).

	TC			Diaphragm		
		
	Normal	Affected		Normal	Affected	
				
Developmental	-	-	+	-	-	+
Fast	265 (73%)	85 (40%)	155 (70%)	222 (48%)	12 (1%)	20 (3.6%)
Fast & slow	0 (0%)	38 (18%)	67 (30%)	0 (0%)	160 (16%)	370 (66.8%)
Slow	96 (27%)	88 (42%)	1 (0%)	243 (52%)	847 (83%)	164 (29.6%)
Total	361 (100%)	211 (49%)	223 (51%)	465 (100%)	1019 (65%)	554 (35%)

The numbers of fibers analyzed were results from a normal or an affected dog. MHC expression between two groups (normal, dMHC (-) *vs *affected, dMHC (-); affected, dMHC (-) *vs *affected, dMHC (+)), or between muscles (TC muscle *vs *diaphragm) was analyzed by Yates's chi-square test. Significant differences (*p *< 0.05) were detected in all tests.

In regenerating fibers of both affected muscles, the distributions of all three populations shifted to smaller sizes than those in the normal counterparts, and a large number of hybrid fibers co-expressing fast and slow type MHCs were observed at a high rate (Fig. 2C and Table 1). In addition, fast MHC fibers were predominant in a regenerating population in TC muscle, while slow MHC fibers were predominant in the diaphragm except for hybrid fibers. These observations suggested that fast fibers could be more susceptible to dystrophic stress than slow fibers, and alteration of MHC expression and regeneration of muscle fibers would be different between TC muscle and the diaphragm.

### Time courses of histology and MHC expression

**Figure 3:**
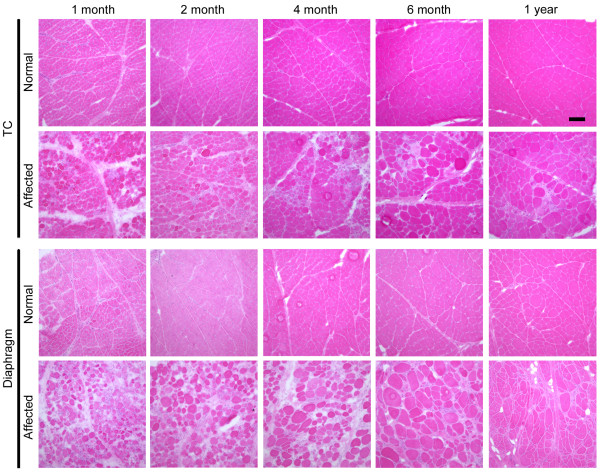
**Representative histological findings in TC muscles and diaphragms of a normal or a CXMD_J _dog at 1, 2, 4, 6 months, and 1 year old**. Note that severe degenerative lesions were observed from early ages in affected diaphragms, as compared with affected TC muscles. Bar: 200 μm.

**Figure 4:**
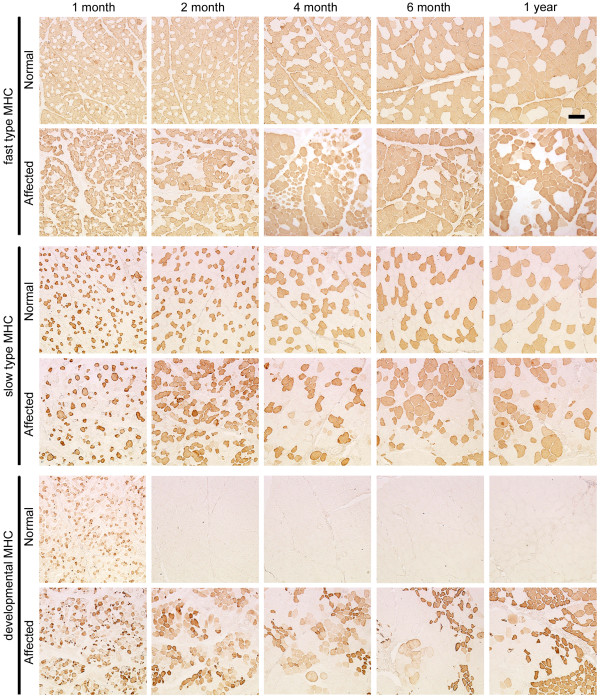
**Expression of fast type, slow type, and developmental MHCs in TC muscles of a normal or a CXMD_J _dog at 1, 2, 4, 6 months, and 1 year old**. Note that there were no notable differences between expression levels of fast and slow type MHCs in normal and affected TC muscles. Bar: 200 μm.

**Figure 5:**
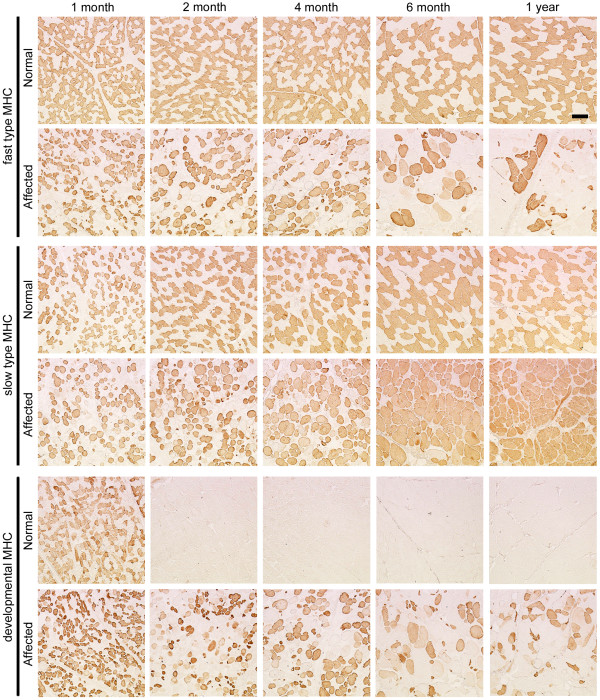
**Expression of fast type, slow type, and developmental MHCs in diaphragms of a normal or a CXMD_J _dog at 1, 2, 4, 6 months, and 1 year old**. Note that slow MHC fibers were increased markedly in the affected diaphragms after 6 months old, while fast MHC fibers were decreased. Bar: 200 μm.

To investigate how MHC expression alters together with growth of CXMD_J_, we examined MHC expression in TC muscles and diaphragms of a normal or an affected littermate at various ages from neonatal to adult stages (1 month to 1 year old) in relation to histopathological features. Affected TC muscles showed mild lesions at 1 and 2 months old, but severe degenerative lesions were evident at over 4 months old (Fig. 3). Expression of fast or slow type MHC did not alter much with aging, and developmental MHC was expressed continuously (Fig. 4). In contrast, degenerative lesions were severe in the affected diaphragm at all ages examined (from 1 month old onward), and endomysial fibrosis was dominantly present over 6 months old (Fig. 3). Fast MHC fiber number decreased markedly, while the number of slow MHC fibers increased significantly in affected diaphragms after 6 months old (Fig. 5). In addition, expression of developmental MHC decreased at 6 months and 1 year old. These observations indicated that MHC expression is altered greatly in the affected diaphragms after 6 months old, unlike TC muscles.

**Figure 6:**
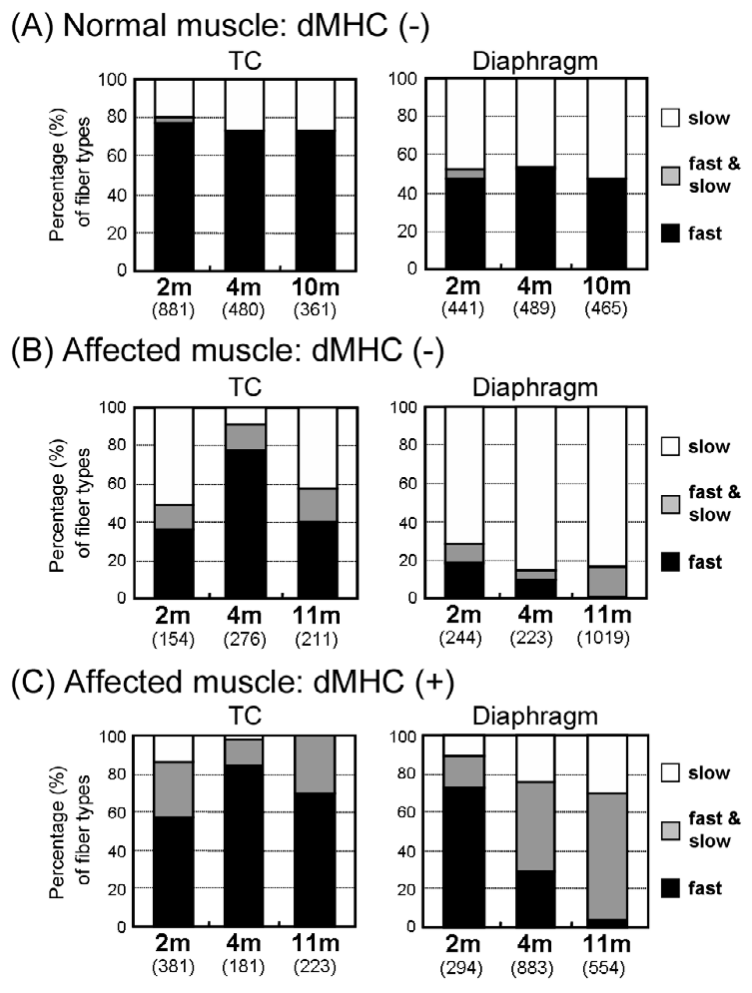
**Proportions of fiber types in skeletal muscles of a normal or a CXMD_J _dog at various ages**. The numbers of fast (black), hybrid (gray), and slow MHC myofibers (white) among populations of myofibers without developmental MHC (A, B) and with developmental MHC (C) were counted in TC muscle and diaphragm of a normal (A) or an affected dog (B, C) at adolescent (2 or 4 months old) or adult stages (10 or 11 months old). The numbers under the ages show total fibers examined. MHC expression between two groups (normal, dMHC (-) *vs *affected, dMHC (-); affected, dMHC (-) *vs *affected, dMHC (+)), between muscles (TC muscle *vs *diaphragm), or among ages (2, 4, and 10 or 11 months) was analyzed by Yates's chi-square test. Significant differences (*p *< 0.05) were detected in all tests, except for no significant differences between 4 and 10 months old in normal TC muscles or diaphragms. Note that slow MHC fibers were consistently larger than other fibers, in populations of muscle fibers without developmental MHC of affected diaphragms. In populations of muscle fibers co-expressing developmental MHC and other MHC isoform(s), slow MHC and hybrid fibers were increased markedly in the affected diaphragm at 4 and 11 months old, unlike TC muscles.

For quantitative evaluation of MHC expression in individual myofibers, we counted three types of MHC-expressing fibers among non-regenerating or regenerating populations within an area in the TC muscle or diaphragm of a normal or an affected littermate (Fig. 6). As normal muscles still expressed developmental MHC at 1 month old (Fig. 4 and 5), we performed the examinations at both adolescent (2 and 4 months old) and adult stages (10 or 11 months old). In normal dogs, the number of fast MHC fibers in TC muscle was three times greater than that of slow MHC fibers throughout aging, while the proportions in the diaphragms remained constant and equivalent between the two types (Fig. 6A). In non-regenerating fibers, the proportions of fiber types were not constant in affected TC muscles at the ages examined, but the majority of these fibers consisted of slow MHC fibers in the affected diaphragms (Fig. 6B). These observations indicated that slow fibers were already predominant in non-regenerating populations of CXMD_J _diaphragms at younger ages. In regenerating fibers, in contrast to the observation that fast MHC fibers consistently accounted for the majority of fibers in affected TC muscles, the affected diaphragms were mainly composed of hybrid and slow MHC fibers and the proportion increased gradually with age (Fig. 6C). These observations indicated that MHC expression in regenerating fibers was also different between affected TC muscle and diaphragm after 4 months old, although it was relatively similar in the two at 2 months old.

### Temporal changes of MHC isoforms

**Figure 7:**
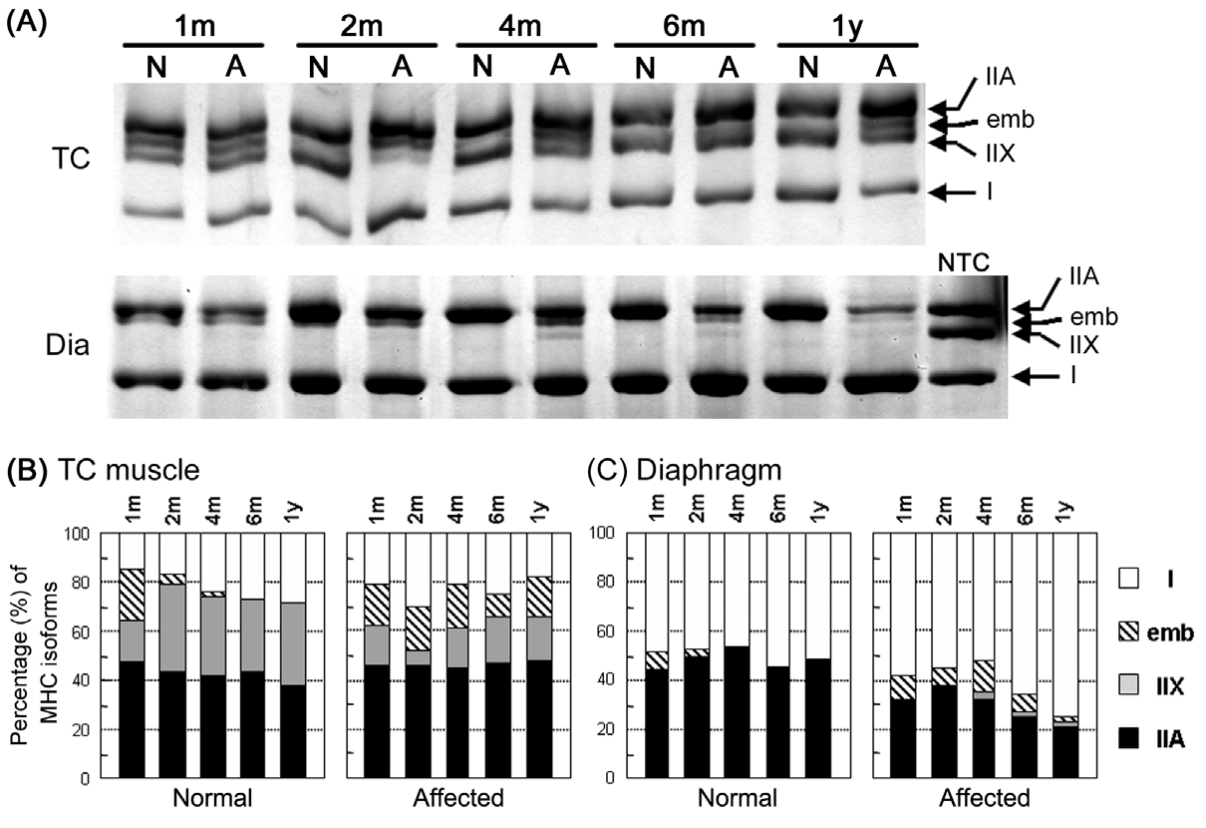
**MHC isoforms in skeletal muscles of normal and CXMD_J _dogs**. (A) Electrophoretic separation of MHC isoforms in TC muscle and diaphragm. Myosin was extracted from muscles at various ages (1, 2, 4, 6 months, and 1 year old), and aliquots of 0.4 μg of protein were separated on 8% SDS-polyacrylamide gels containing 30% glycerol. Four MHC isoforms (I, IIX, IIA, and embryonic) were detected. NTC: normal TC muscle at 1 year old. Note that MHC type I increased in the affected diaphragm after 6 months old. (B) Quantitative analysis of MHC isoforms. MHC expression between two groups (normal *vs *affected) or among ages (1, 2, 4, 6 months and 1 year) was analyzed by Yates's chi-square test. Significant differences (*p *< 0.05) were detected between normal and affected groups in TC muscles after 2 months old or in diaphragms after 4 months old, and between 1 and 2 months old in normal TC muscles.

To examine how progressive degeneration alters the composition of fiber types in affected skeletal muscles, we detected myosin isoforms in TC muscles and diaphragms of CXMD_J _at various ages by electrophoretic gel separation (Fig. 7). Four MHC isoforms (I, IIA, IIX, and embryonic), which migrated on electrophoresis as IIA-embryonic-IIX-I from slowest to fastest [11,12], were detected in canine skeletal muscles (Fig. 7A). In affected TC muscles, type I, IIA, and embryonic isoforms were consistently detected at similar levels, but the level of type IIX MHC was lower than those in normal TC muscles after 2 months old. In contrast, type IIA MHC level decreased gradually in affected diaphragms with growth, and type I accounted for the majority of MHC components in animals over 6 months old. In addition, the embryonic isoform decreased in affected diaphragms after 6 months old. These results were consistent with those of immunohistochemical analyses (Figs. 4 and 5). These observations suggested that type IIX and IIA fast fibers may be preferentially affected in TC muscle and diaphragm of CXMD_J_, respectively. Furthermore, these observations suggested that muscle regeneration may deteriorate from relatively younger age in the affected diaphragm, unlike TC muscle.

## Discussion

To investigate the alterations in fiber types in skeletal muscles of a canine DMD model, we examined MHC expression in the TC muscle and diaphragm of CXMD_J _at various ages. Our results indicated that the influences of dystrophin deficiency on fiber type composition were significantly different between TC muscle and diaphragm.

To analyze MHC expression in details, we compared fiber type composition and fiber size distribution of MHC-expressing fibers between a normal dog (10 months old) and an affected dog (11 months old). In normal and affected dogs, body weight rapidly increased to approximately 9 kg at 4 months old, and then slightly increased to approximately 14 and 11 kg at 12 months old, respectively [5]. As body weight reflects muscle weight, muscle mass and fiber size would not extremely change in 1 month after 4 months old, especially in normal dogs. In fact, in TC muscles or diaphragms of normal dogs, there were no significant differences among compositions of fiber types and MHC isoforms after 4 months old (Fig 6 and 7). In addition, we examined normal dogs at 11, 12 and 14 months old, and affected dogs at 10, 12, 13 and 15 months old. Normal muscles of adult dogs showed similar expression of fast type, slow type, or developmental MHC at all adult ages, and affected muscles also showed similar MHC expression at examined ages (data not shown). These observations implied that there would be no significant difference in MHC expression between at 10 and 11 months old, in both of normal and affected dogs.

### Common features between TC muscle and diaphragm of CXMD_J_

TC muscle and diaphragm of CXMD_J _shared the features that slow MHC fibers increased and enlarged selectively in non-regenerating populations, while fast type IIX or IIA MHC isoform decreased. Similar observations have been reported in skeletal muscles of the *mdx *mouse [13], GRMD [14], and human DMD [12,21]. In general, increasing and enlarging of slow fibers may be a consequence of adaptive responses by metabolic enzyme systems and energy consumption, because slow fibers have lower capacity for power output and consume less energy than fast fibers [22]. Our results also supported the hypothesis that slow fibers would be more adaptable to dystrophic stress than fast fibers, to compensate for the reduced abilities of muscle function.

Two mechanisms were considered to explain the selective increase in slow fibers during progressive muscle degeneration. One possibility is that slow fibers may be more resistant to dystrophic stress than fast fibers, leading to selective survival of slow fibers. This was supported by the observation that slower muscle fibers contained significantly more utrophin, a homolog of dystrophin, in comparison to faster counterparts [23,24]. Another is transition of MHC isoforms, where type IIA or IIX MHC isoforms could be transited to type I, as seen in hypertrophy and exercise [25]. MHC I, IIa, IIx, and IIb gene expression are known to be regulated by the calcineurin pathway [26,27]. Dystrophin deficiency may accelerate MHC transition to slower types *via *calcineurin/NFAT signaling in skeletal muscles of CXMD_J_, because calcineurin and activated NFATc1 protein content were higher in muscles from *mdx *than wild-type mice [28]. However, it remains possible that both mechanisms may be active at the same time, because the calcineurin/NFAT cascade can regulate not only the MHC promoters but also the utrophin A promoter [24,29,30].

### Differences between TC muscle and diaphragm of CXMD_J_

The CXMD_J _diaphragm developed severe degenerative lesions from earlier stages than TC muscle, which corresponded to previous reports [3,5,31]. In addition, dystrophic changes in the CXMD_J _diaphragm not only markedly altered the expression of fast and slow type MHCs but also decreased the amount of the developmental (embryonic and/or neonatal) MHC with growth, unlike affected TC muscle. Especially, fast MHC fibers disappeared and slow MHC fibers enlarged in the adult CXMD_J _diaphragm. The greater cross-sectional area of slow fibers in affected diaphragms might be due to hypertrophy in compensation for loss of fast fibers, relating to plasticity of muscle fibers, as mentioned above. The diaphragm keeps continuous contraction of muscle fibers without resting, while limb skeletal muscle regularly rests its movement. Therefore, replacement with slow fibers may be particularly enhanced in the diaphragm rather than TC muscle, depending on pathological severity and contractile activity of skeletal muscles. 

Fiber type determination and fiber type-specific gene expression are regulated by multiple signaling pathways and transcription factors. As partially described above, a key mediator, calcineurin, plays an important role in acquisition of fiber phenotype [29,30] and may induce not only transition of MHC isoforms from faster to slower types but also transformation of myofiber phenotypes in mouse or rat muscles [26,27,32]. In addition, calcineurin signaling activity was greater in the diaphragm than in the tibialis anterior muscle of the *mdx *mouse [28]. Therefore, replacement with slow fibers may be up-regulated to a greater extent in the diaphragm than in the TC muscle of CXMD_J_.

We also showed age-related changes of MHC expression in affected diaphragms after 6 months old, in contrast to TC muscles (Fig 4, 5 and 7). In addition, fiber type compositions in non-regenerating or regenerating fibers were also different between the TC muscle and the diaphragm, depending on age. In non-regenerating fibers of affected TC muscles, fast MHC fibers at 4 months old was higher than those at 2 and 11 months old (Fig. 6B). It might be partially involved in pathological changes that degenerative lesions appeared obviously in affected TC muscles after 4 months old, as described previously [3,5,31]. In regenerating fibers of the CXMD_J _diaphragm, the proportion of myofibers expressing slow type MHC increased markedly after 4 months old (Fig. 6C). These results suggested that MHC expression in TC muscle and the diaphragm of CXMD_J _would be influenced by different mechanisms after 4 months old. These age-dependent MHC expression might be related to body growth, particularly increasing of muscle mass. One possibility is participitation of insulin-like growth factor (IGF)-1, which is important for postnatal growth of skeletal muscles [33] and can activate multiple Ca^2+^-dependent signaling pathways, including the calcineurin/NFAT pathway [30]. When growth rate of body weight decreases after 4 months old [5], signaling activity of IGF-1 might reduce and MHC expression might be regulated predominantly by alternative signaling pathways.

### Comparison among *mdx*, CXMD_J_, and DMD diaphragms

MHC expression in normal skeletal muscle has been well studied in mice [15,34], dogs [11], and humans [35]. In normal dogs, the proportions of fiber types in TC muscle were relatively similar to those in the representative tibialis anterior muscles of mice and humans. In the diaphragm, however, the proportion of fiber types differed markedly among these species. The murine diaphragm is composed mainly of fast type IIA and IIX isoforms [15,34], but the canine diaphragm consists of equal populations of slow type MHC I and fast type MHC IIA [11], as also shown in our study. In normal human diaphragm, the distribution of myosin isoforms has been estimated that types I, IIA, and IIX account for approximately 45%, 40%, and 15%, respectively [35]. Thus, the proportions of MHC isoforms in the diaphragm of healthy dogs are much closer to those of humans than those of mice.

Some groups have studied expression profiles of MHC isoforms in the diaphragm of the *mdx *mouse. The *mdx *diaphragm shows increases in MHC type I fibers and elimination of type IIX population at 2 years old, but not at young ages (3 to 6 months old) [13,15,34]. In contrast to the *mdx *diaphragm, that in CXMD_J _exhibited drastic changes even in younger animals (6 months old). On the other hand, there is no direct information available regarding the changes in fiber type composition in the diaphragm in human DMD. In addition, there is an important difference of MHC expression even in limb skeletal muscles between large mammals (including dogs and humans) and mammals with smaller body mass, especially rodents. The former do not express the fastest MHC IIB isoform in limb muscles [10,11,36], while it is abundantly expressed in the latter [34]. Therefore, changes/adaptations in skeletal muscles of dogs with muscular dystrophy are likely to be more relevant to human DMD, than that in the *mdx *mouse. As it is difficult to examine the diaphragms of DMD patients, it would be important to investigate the differences between murine and canine models for understanding the mechanisms of respiratory failure in human DMD.

## Conclusion

Based on fiber type classification using MHC expression, we demonstrated the predominant replacement with slow fibers and reduced muscle regeneration with progression of muscular dystrophy in the diaphragm of a canine DMD model, but these phenomena were much less strict in affected TC muscle. In addition, the expression profiles of MHC isoforms in the CXMD_J _diaphragm were evidently different from those of the *mdx *mouse. Our results indicated that dystrophic dog is a more appropriate model than a murine one for human DMD, and would be useful for investigation of the mechanisms of respiratory failure in DMD, as well as pathological and molecular biological backgrounds, and therapeutic effects in clinical trials.

## Competing interests

The author(s) declare that they have no competing interests.

## Authors' contributions

KY designed the study, carried out the pathological and immunohistological examinations, and drafted the manuscript. AN participated in interpretation of data, and helped to draft the manuscript. TH participated in coordination of the study. ST participated in the design, planning, and coordination of the study, and helped to draft the manuscript. All authors read and approved the final manuscript.

## Pre-publication history

The pre-publication history for this paper can be accessed here:


